# Providing solid angle formalism for skyshine calculations

**DOI:** 10.1120/jacmp.v11i4.3286

**Published:** 2010-08-17

**Authors:** Michael S. Gossman, A. Jussi Pahikkala, Mary B. Rising, Patton H. McGinley

**Affiliations:** ^1^ Tri‐State Regional Cancer Center Medical Physics Section Ashland KY USA; ^2^ FI‐21310 Vahto Finland; ^3^ Department of Mathematics University of Louisville Louisville KY USA; ^4^ 685 Tahoe Circle Stone Mountain GA 30083 USA

**Keywords:** scattering, shielding, skyshine, solid angle

## Abstract

We detail, derive and correct the technical use of the solid angle variable identified in formal guidance that relates skyshine calculations to dose‐equivalent rate. We further recommend it for use with all National Council on Radiation Protection and Measurements (NCRP), Institute of Physics and Engineering in Medicine (IPEM) and similar reports documented. In general, for beams of identical width which have different resulting areas, within ±1.0% maximum deviation the analytical pyramidal solution is 1.27 times greater than a misapplied analytical conical solution through all field sizes up to $40\times 40\;{cm}^{2}$. Therefore, we recommend determining the exact results with the analytical pyramidal solution for square beams and the analytical conical solution for circular beams.

PACS number(s): 87.52.‐g, 87.52.Df, 87.52.Tr, 87.53.‐j, 87.53.Bn, 87.53.Dq, 87.66.‐a, 89., 89.60.+x

## I. INTRODUCTION

The determination of skyshine involves the calculation of the solid angle subtended by a linac radiation beam of known field size and square shape. Previous research has been documented where analytical conical expressions were used.^(^ ^1^ ^–^ ^3^ ^)^ Cone‐based equations are more appropriate for circular collimation such as from round apertures of cerrobend blocks. An analytical pyramidal equation should be used when medical accelerators are involved, since square‐shaped apertures result in an inverted pyramid shaped beam. We detail, derive and correct the technical use of the solid angle variable identified in formal guidance that relates skyshine calculations to dose‐equivalent rate. We further recommend it for use with all NCRP, IPEM and similar reports documented.^(^ ^1^ ^–^ ^4^ ^–^ ^8^ ^)^

## II. MATERIALS AND METHODS

McGinley^(^ ^2^ ^)^ has shown that the skyshine measured dose‐equivalent rate $\dot{H}\;(\text{nSv}\;h^{‐1})$ is directly dependent on the transmission through the barrier. The form of the equation is shown in Eq.1:

(1) $$\dot{H}=\frac{2.5\times 10^{7}\left(B_{xs}{\dot{D}}_{o}\Omega^{1.3}\right)}{{\left(d_{i}d_{s}\right)}^{2}}$$

where ${\dot{D}}_{o}\;(\text{Gy}/h)$ is the X‐ray absorbed dose‐rate at 1 m from the target, $d_{i}$ (m) is the vertical distance from the target to a point 2 m above the roof, $d_{s}$ (m) is the lateral distance from the isocenter to a point outside the barrier where measurements are to be taken, Ω is the solid angle formed by the radiation beam in steradians and is given by Eq. 2:

(2) Ω=2π(1‐cosθ)

In this equation, the angle Θ (degrees) is the angle subtended between the central axis and the edge of the beam as radiation projects away from the source. Thus, Eq. 2 represents the analytical conical expression, where the beam pointing upward would resemble an inverted cone.

Equation 2 represents the correct form of the solid angle equation, but only for circular fields such as from those formed by cerrobend blocks. It is rarely considered how the effect of cerrobend blocking with circular apertures affects radiation levels outside the vault. It is nearly universal that accelerators include moving jaw systems or even multileaf collimators, which may be completely opened to a square dimension as large as $40\times 40{\text{cm}}^{2}$ defined at 1m from the machine isocenter. It is these square apertures that are used for radiation protection purposes. For discussion here, we define the distance *h* as the height of the beam for which the solid angle is determined, commonly used as the 100 cm source‐axis‐distance (SAD) for accelerators, where the field size is defined. Given this geometry, the radiation beam will no longer resemble a cone, but rather an inverted pyramid with SAD‐projected field size denoted *a*. The form of the resulting equation for the solid angle, which should be used for all medical accelerator skyshine calculations, is expressed in Eq. 3:

(3) $$\Omega =4\arcsin\frac{a^{2}}{a^{2}+4h^{2}}$$

## III. RESULTS & DISCUSSION

We present an accurate solid angle equation necessary to considering skyshine mathematically. A derivation is presented in the Appendix to this research for the correct form of the solid angle. An analysis of the numerical change expected while using it instead of the conical expression is also discussed, to prevent further inconsistencies found elsewhere in literature.

For the purposes of shielding, only perfectly square fields should be used. A resulting solid angle for the $40\times 40{\text{cm}}^{2}$ field size (a=40) defined at a distance of 100 cm (h=100) is thus Ω=0.1539 steradians. It is noteworthy for approximating results that, for a typical square clinical aperture, the analytical conical solution will give good approximations (within 0.2% of Eq. 3) for the solid angle, but only when Θ is determined such that the area of the circular base is equal to the square aperture area. Therefore, an approximated solution, though inexact, is achievable by comparing the resulting $1600\;{\text{cm}}^{2}$ square area to the circular area given as πr^2^. The resulting equivalent circular radius denoted *r* (cm), which is also equivalent to the quotient α/2 representing half the length of the field, is then found to be $40\pi^{-1/2}$ cm or 22.6 cm. The conical projection with Θ=0.2220 radians (12.7°) for this geometry results in a solid angle of Ω=0.1541 1steradians. This is very comparable to the pyramidal solution, although inexact.

Still, with the solid angle dependence varying as $\Omega^{13}$, skyshine dose‐equivalent rate error increases dramatically when equations are misused. Thus, it is very important to simplify calculations whenever possible. One cannot assume the same base width (diameter) for the circular beam as for the square beam. In general, for beams of identical width which have different resulting areas, within ±1.0% maximum deviation the analytical pyramidal solution is 1.27 times greater than the analytical conical solution through all field sizes up to $40\times 40{\text{cm}}^{2}$. Therefore, we recommend determining the exact results with the analytical pyramidal solution for square beams and the analytical conical solution for circular beams. Results for 6 MV and 18 MV X‐rays are already published.^(^ ^3^ ^,^ ^9^ ^)^

## Figures and Tables

**Figure 1:**
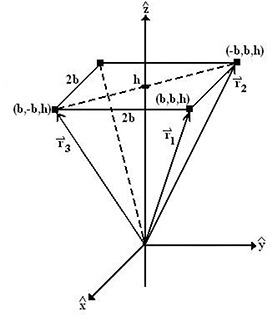
Simplification of the projection of a modern linear accelerator beam having a square type projection as it relates to the variables used in this derivation.

**Figure 2:**
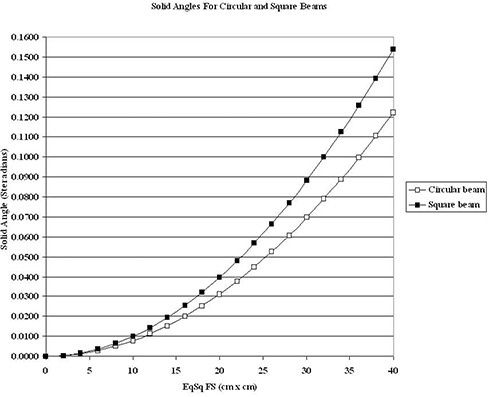
Apical solid angle comparison for a radiation field defined by a square beam (using the exact formula for an inverted pyramid), and for the circular beam in Eq. 2 where the diameter is inappropriately approximated as the side of the square pyramidal field.

## Appendix

For a summary of the derivation of the analytical pyramidal solid angle solution, consider a right pyramid with a square‐formed base with sides a=2b and height *h* (Fig. 1). Here, the pyramid is set into the x, y, z coordinate system so that its apex is at the origin (0, 0, 0), with the center of the base on the z‐axis and the sides of the base parallel to the x‐ and y‐axes. The four vertices of the base are (±b,±b,h) Considering only the three vectors pointing to (*b,b,h*), ((−b,b,h)) and (b,−b,h), let the position vectors (i.e. the vectors from the origin to these points) be ${\vec{r}}_{1},{\vec{r}}_{2}$ and ${\vec{r}}_{3}$, respectively. We begin by starting with the relation

(4) $$\tan\frac{\Omega^{\prime }}{2}=\frac{\left({\vec{r}}_{1}{\vec{r}}_{2}{\vec{r}}_{3}\right)}{{\vec{r}}_{1}•{\vec{r}}_{2}{\vec{r}}_{3}+{\vec{r}}_{2}•{\vec{r}}_{3}{\vec{r}}_{1}+{\vec{r}}_{3}•{\vec{r}}_{1}{\vec{r}}_{2}+{\vec{r}}_{1}{\vec{r}}_{2}{\vec{r}}_{3}}$$

as described by Oosterom and Strackee,^(^ ^10^ ^)^ which presents half of the whole apical solid angle Ω' of the pyramid determined by the vectors ${\vec{r}}_{1},{\vec{r}}_{2},{\vec{r}}_{3}$. The numerator of the right‐hand side is the scalar triple product of the three vectors. Now the three position vectors are in the component form

(5) $${\vec{r}}_{1}=(b,b,h),\;\;{\vec{r}}_{2}=(‐b,b,h),\;\;\;{\vec{r}}_{3}=(b,‐b,h).$$

Thus their scalar triple product in the numerator is

(6) $$\left|\begin{array}{lll} r_{1x} & r_{1y} & r_{1z}\\ r_{2x} & r_{2y} & r_{2z}\\ r_{3x} & r_{3y} & r_{3z} \end{array}\right|=(r_{1x},r_{2y}‐r_{2x}r_{1y})r_{3z}+(r_{1y},r_{2z}‐r_{2y}r_{1z})r_{3x}+(r_{1z},r_{2x}‐r_{2z}r_{1x})r_{3y}$$

resulting in

(7) $$({\vec{r}}_{1}{\vec{r}}_{2}{\vec{r}}_{3})=(bb‐(‐b)b)h+(bh‐bh)b+(h(‐b)‐hb)(‐b)=4b^{2}h=a^{2}h.$$

The lengths of all three vectors are the same such that

(8) $$r=r_{1}=r_{2}=r_{3}=\sqrt{b^{2}+b^{2}+h^{2}}=\sqrt{2b^{2}+h^{2}}$$

is the common factor in all four terms of the denominator. It may be calculated as

(9) $$({\vec{r}}_{1}\cdot {\vec{r}}_{2}+{\vec{r}}_{2}\cdot {\vec{r}}_{3}+{\vec{r}}_{3}\cdot {\vec{r}}_{1}+r^{2})r$$

which factors out as

(10) $$[h^{2}+(‐2b^{2}+h^{2})+h^{2}+{\left(\sqrt{2b^{2}+h^{2}}\right)}^{2}]\sqrt{2b^{2}+h^{2}}=4h^{2}\sqrt{2b^{2}+h^{2}},$$

and where with the substitution $b=\frac{a}{2}$, the final value of the denominator results in $2h^{2}\sqrt{2a^{2}+4h^{2}}$. Accordingly, we can substitute to the formula (11) the values of numerator and denominator, obtaining

(11) $$\tan\frac{\Omega^{\prime }}{2}=\frac{a^{2}h}{2h^{2}\sqrt{2a^{2}+4h^{2}}}=\frac{a^{2}}{2h\sqrt{2a^{2}+4h^{2}}}.$$

The result becomes simpler, if we transfer from the tangent to the sine; there exists for acute angles the trigonometric formula $\alpha \frac{\tan\alpha }{\sqrt{1+\tan^{2}\alpha }}$, where it is seen that

(12) $$\sin\frac{\Omega^{\prime }}{2}=\frac{a^{2}}{a^{2}+4h^{2}}$$

Again, since we use only three vertices of the base, the formula yields half of the wanted solid angle. The whole apical solid angle $\left(\Omega =2\times \Omega^{\prime }\right)$ in its simplest form is then exactly as presented in Eq. 3, where *h* is the distance for which the pyramidal shape is defined as

(3,13) $$\Omega =4\arcsin\frac{a^{2}}{a^{2}+4h^{2}}$$

Figure 2 shows a plot comparison of the subtended apical solid angle for both solution types as a function of aperture field diameter.
